# Epidemiology, patterns, and mechanisms of pediatric trauma: a review of 12,508 patients

**DOI:** 10.1007/s00068-022-02088-6

**Published:** 2022-08-24

**Authors:** Raffael Cintean, Alexander Eickhoff, Jasmin Zieger, Florian Gebhard, Konrad Schütze

**Affiliations:** Department of Trauma-, Hand-, and Reconstructive Surgery, Ulm University, Albert-Einstein-Allee 23, 89081 Ulm, Germany

**Keywords:** Pediatric injury, Mechanism

## Abstract

**Background:**

Pediatric traumas are common and remain a unique challenge for trauma surgeons. Demographic data provide a crucial source of information to better understand mechanisms and patterns of injury. The aim of this study was to provide this information to improve treatment strategies of potentially preventable morbidity and mortality in children.

**Material and methods:**

A retrospective review of every pediatric trauma treated in the emergency department (ED) between 2015 and 2019 was performed. Inclusion criteria were the age between 0 and 14 years and admission to the ED after trauma. Demographic data, time of presentation, mechanism of injury and pattern of injury, treatment, and outcome were analyzed. Different injury patterns were assessed in relation to age group, sex, mechanism of injury and treatment.

**Results:**

A total of 12,508 patients were included in this study. All patients were stratified into five age groups: babies under the age of 1 (8.8%), toddlers between 1 and 3 (16.8%), preschool children between 4 and 6 (19.3%), young school children between 7 and 10 (27.1%), and young adolescents between 11 and 14 (27.9%). The predominant sex in all age groups was male. 47.7% of patients were admitted between 4 and 10 pm; 14.8% of the patients arrived between 10 pm and 8 am. Peak months of admissions were May to July. Overall, 2703 fractures, 2924 lacerations and superficial tissue injury, 5151 bruises, 320 joint dislocations, 1284 distortions, 76 burns, and 50 other injuries were treated. Most common mechanisms for fractures were leisure activities, falls, and sports-related activities. Forearm fractures were the most common fractures (39.5%) followed by humerus fractures (14%) and fractures of the hand (12.5%). A total of 700 patients with fractures (25.9%) needed surgery. 8.8% of all patients were hospitalized for at least one day. 4 patients died in the hospital (0.03%).

**Conclusion:**

Despite of higher risk, severe injuries in children are rare. Minor injuries and single fractures are common. Treatment should be managed in specialized centers to ensure an interdisciplinary care and fast recovery. Peak times in the late afternoon and evening and summer months should be taken into consideration of personnel planning.

## Introduction

Unintentional injuries are a significant health risk for children and adolescents. According to estimates by the World Health Organization, more than 830,000 under18s are killed in accidents worldwide every year [1]. In Germany, accident-related deaths among children and adolescents have been declining for years, but in 2020 traumatic injuries were still the second most common cause of death among children aged 1 to 15 [2]. Injuries in childhood and adolescence are also a frequent cause of hospitalization. According to the German Federal Statistical Office, accidents are the most common cause of hospitalization between the ages of 5 and 19 and the second most common cause between the ages of 1 and 4 [3].

The morbidity and mortality of children involved in accidents not only pose great challenges for those treating them but also long-term consequences, like physiological and economic aspects, must be taken into account [4–6].

Numerous studies report incidence and mechanism of severely injured children [7–11]. However, only few reports are found on all children requiring treatment for injury [12–14].

This study was done to provide important information on the incidence, type, and mechanism of injury in children in order to help in developing prevention strategies.

## Methods

This study was a retrospective exploratory review at a Level One Trauma Center in Germany. Every patient between 0 and 14 years of age who was treated for a trauma in our emergency department (ED) between January 2015 and December 2019 was identified and included in this study. Exclusion criteria were only patient age over 14 years and admission to the ED due to conditions other than trauma.

Patient demographics, time and month of presentation, type and mechanism of injury, and need for surgical care were examined. Patients were stratified in age groups babies up to 1, toddlers between 1 and 3, preschoolers between 4 and 6, young school children from 7 to 10, and young adolescents between 11 and 14 years of age. All mechanisms of injury were recorded and classified. Injuries were classified as fractures, distortions, lacerations, joint dislocations, bruises, burns, and multiple injuries or others. The exact body regions were recorded for all injuries. All injuries were analyzed in terms of mechanism, necessity of surgical treatment, and time of presentation in the ED.

The mechanisms of injury were classified into 8 categories:

- Leisure activity
  Leisure activities include mechanisms that could not be assigned to a sport or other mechanism. Included were general play, walking and running, accidents on the playground, and accidents at home.
- Sports-related activity
  Sports-related activities include all team sports, such as soccer, basketball, or handball. Also, winter sports, like skiing, snowboarding, and ice skating, were included.
- Falls
  Falls include all patients who have fallen from objects. These include beds, couches, chairs, climbing frames, etc. Falls from standing or walking were listed under the categories leisure activity or sports-related activity.
- Blunt trauma/collision
  Blunt trauma includes any blows or impacts as well as collisions with other people or objects.
- Road traffic accidents (RTAs)
  RTAs include all accidents that have occurred on the road. This includes accidents as a passenger in a car or on a motorcycle as well as passengers on public transport. In addition, all accidents that occurred as pedestrians or cyclists in road traffic were included.
- Violence
  Violence includes all acts of violence, such as violence among persons or acts of violence with objects or weapons.
- Cutting/stabbing
  Cutting and stabbing mechanisms include all injuries by sharp objects.
- Burns
  Burns include all superficial and deep burns caused by warm or hot objects and liquids. (Table 1).

**Table 1:** Mechanisms of injury by age groups

Mechanism	< 1 years	1–3 years	4–6 years	7–10 years	11–14 years	All
Leisure activity	392	521	614	712	623	2862
Sports-related activity	15	212	311	892	1234	2664
Falls	281	513	524	589	504	2411
Blunt trauma	298	332	313	453	512	1908
Road traffic accident	9	231	312	321	265	1138
Force/violence	14	151	115	181	130	591
Cutting/stabbing	0	67	104	141	105	417
Burns	11	19	21	18	7	76
Misc/no documentation	83	61	101	87	109	441

## Results

**Fig. 1:**
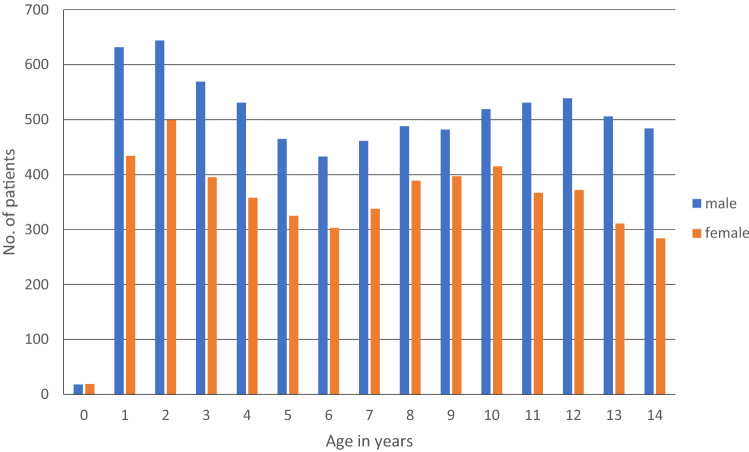
Distribution of age and gender

A total of 12,508 patients were included in this study, of which 7302 were male and 5206 female. In all age groups, most patients were male. The age group with the most patients was young teenagers between 11 and 14 with a total of 3489 patients (27.9%). The demographic data are shown in Table 2. The age group distribution shows two peaks at a very young age between 1 and 3 years and again between 11 and 14 years (Fig. 1).

**Table 2:** Patients demographics

Patients demographics	
Sex	
Male	7302 (58.4%)
Female	5206 (41.4%)
Age distribution	
< 1 year	1103 (8.8%)
1–3 years	2107 (16.8%)
4–6 years	2415 (19.3%)
7–10 years	3394 (27.1%)
11–14 years	3489 (27.9%)
Type of injury	
Bruise	5151 (41.2%)
Laceration	2924 (23.4%)
Fracture	2703 (21.6%)
Distortion	1284 (10.3%)
Joint dislocation	320 (2.6%)
Burns	76 (0.6%)
Polytrauma/other	50 (0.4%)
Mechanism of injury	
Leisure activity	2862 (22.9%)
Sports-related activity	2664 (21.3%)
Falls	2411 (19.3%)
Blunt trauma	1908 (15.3%)
Road traffic accidents	1138 (9.1%)
Force/violence	591 (4.7%)
Cutting/stabbing	417 (3.3%)
Burns	76 (0.6%)
Misc./no documentation	441 (3.5%)

### Injuries and mechanisms

All injuries were classified into 6 categories and further analyzed for the different body regions. Contusions and bruises were the most common type of injury with 5151 cases, followed by lacerations and fractures. Rare traumas as well as multiple injuries were classified under other (Table 3).

**Table 3:** Injuries by age group

Injury	< 1 years	1–3 years	4–6 years	7–10 years	11–14 years	All
Bruise	681	945	1083	1180	1262	5151
Laceration	159	531	519	845	870	2924
Fracture	141	324	551	873	814	2703
Joint dislocation	76	167	12	32	33	320
Distortion	38	128	211	423	484	1284
Other	8	12	39	41	26	126

### Bruises

A total of 5151 children with bruises were treated in our ED. The most common location was the hand with 1137 patients (22.1%) and the head with 867 patients (16.8%).

Most common accident mechanism resulting in bruises were falls (*n* = 1402, 27.2%) followed by sports (*n* = 1099, 21.3%) and leisure activities (*n* = 1098, 21.3%). Patient with bruises after blunt trauma was treated 625 times (12.1%), RTA 576 times (11.2%), and violence 351 times (6.8%).

### Lacerations

**Fig. 2:**
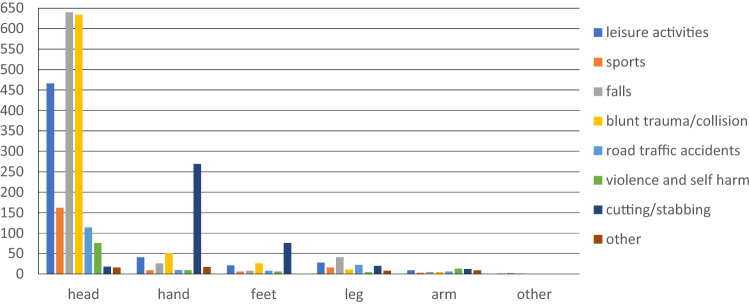
Mechanisms and locations of lacerations

2924 patients suffered a laceration or a wound. 2089 patients (71.4%) were treated for a laceration in the head region, 446 for wounds on the hand (15.3%) and 163 patients for lacerations on the feet (5.6%). Most common mechanism was a blunt trauma or collision (*n* = 727, 24.9%), falls in 721 patients (24.6%) and leisure activities in 566 cases (19,4%) as well as cutting or stabbing injuries in 395 cases (13.5%). 58 (1.9%) patients were treated for animal or human bites (Fig. 2).

### Fractures

**Fig. 3:**
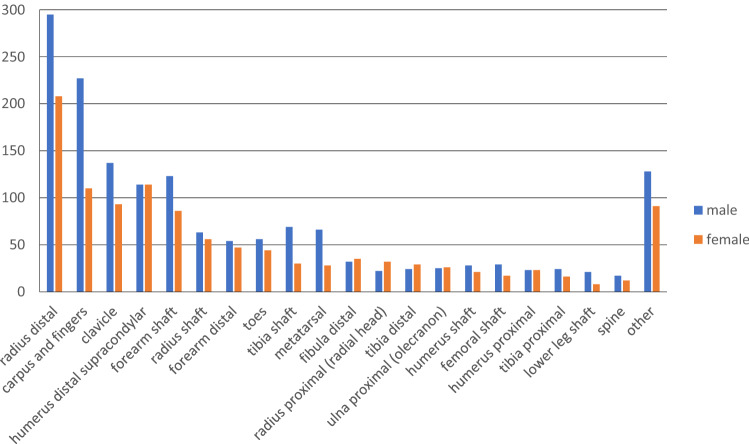
Distribution of locations of fractures

**Fig. 4:**
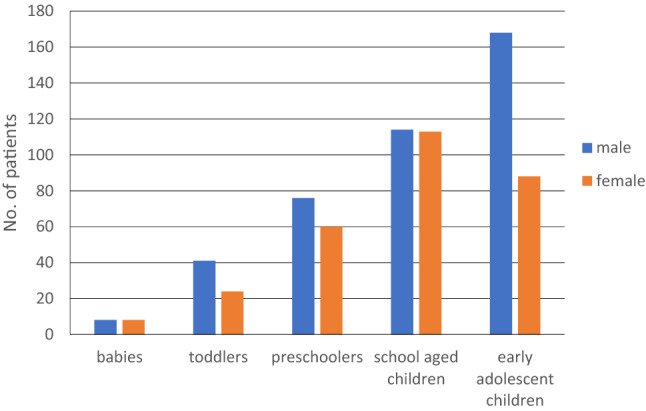
Age distribution of patients with fractures needing surgery

Fractures occurred in 2703 cases. Most common fractures were the distal radial fractures with 503 patients (18.6%), hand and finger fractures with 337 patients (12.5%) and the fracture of the clavicle with 230 patients (8.5%) (Fig. 3) 700 children (25.9%) with fractures needed surgical treatment. The most common injuries requiring surgery were forearm shaft fractures (54.4%), supracondylar humerus fractures (20.1%) as well as lower leg fractures (11.9%). (Table 4) Apart from few exceptions like the distal fibula, radial head or the distal tibia, most patients with fractures were male. In those patients needing surgery, a steady growth in incidence in the age group distribution with a decline in girls in early adolescence could be found (Fig. 4).

**Table 4:** Distribution of fractures needing surgery

Surgery	*n*	%
Forearm fracture	381	54.4
Humerus fracture	141	20.1
Lower leg fracture	83	11.9
Hand fracture	37	5.3
Femur fracture	36	5.1
Foot fracture	11	1.6
Chest fracture	7	1.0
Spine fracture	2	0.3
Cranial bone fracture	2	0.3

Leisure activities (*n* = 872, 32.3%) and falls (*n* = 721, 26.7%) resulted in fractures most commonly, followed by sports-related activities (*n* = 614, 22.7%).

### Distortions

Because of the terminological similarity with bruises in the notes, distortions were divided into only three subcategories. The most frequent distortion was supination trauma of the ankle joint with 1034 patients (80.5%). Cervical spine strain was treated 238 times (18.5%). 12 patients presented with distortion of the knee joint (0.9%). Sports-related activities (*n* = 492, 38.3%) and leisure activities (n = 472, 36.8%) were the most common mechanism for ankle sprains. In sprains of the cervical spine, RTA is second most common mechanism with 61 cases (25.6%).

### Joint dislocations

A total of 320 patients with joint dislocations were treated. Also included were subluxations of the radial head, also known as Nursemaids Elbow, which are very common especially in young children. With 240 patients (75%), this group also accounted for the majority of patients with dislocations. Female children were significantly more likely to be treated for subluxation of the radial head (*p* < 0.05). Other diagnoses were patellar dislocations (13.4%), elbow dislocations (7.5%), AC joint dislocations (3.8%), and shoulder dislocations (0.3%).

Leisure activities and falls were the most common mechanism of the nursemaid’s elbow, which usually happens when preventing a fall by pulling on the arm or playing with the child (75.0%). Further mechanisms for joint dislocations were sports-related activities (*n* = 34, 10.6%). RTA accounted for 2.2% of all joint dislocations.

### Burns

76 patients were treated for burns in our ED. As our hospital is not a certified burn center, the majority of burns were of mild to moderate severity. Children are usually treated at our affiliated children's hospital, which is why the number appears to be rather low. The hands and forearms were most frequently affected (59.2%), followed by the thigh and head with 8 patients each (10.5%). Other localizations were chest (7.9%), feet (7.9%), upper arms (2.6%), and lower legs (1.3%).

Most common mechanism was related to food (hot food, tea, etc.) (53.9%), hot oven or fire place (30.3%), or open fire and firecrackers (15.8%).

### Polytrauma/other

6 children (0.05%) were admitted through our shock room as polytrauma with an Injury Severity Score (ISS) > 16. One child was buried under a stone slab and showed severe chest and mediastinal injuries. One child was hit by a car as a pedestrian and was admitted with a severe brain injury, a pelvic fracture, and femur and forearm fracture. One child showed a pelvic as well as femur and tibia fracture after falling from a horse. Two children were admitted with severe brain injury and mediastinal injuries after an RTA as a pedestrian and cyclist. One child was hit on the head by a streetlight after it was knocked down by a car and showed severe brain injuries as well as multiple scull and midfacial fractures.

In the analyzed period, 4 patients died of an unintentional injury (0.03%). 3 patients died on the day of admission, and one child died on the ICU. All patients died of traumatic brain injuries after a road traffic accident as a pedestrian or cyclist.

In 4 patients, the pattern of injury could not be clearly associated with the accident mechanism, so suspicion of child abuse was raised. All 4 patients showed bruises in different locations. One child was diagnosed with multiple rib fractures as well as a humerus fracture. All patients were presented to the child protection services.

4 patients were admitted due to suicidal attempts. Mean age in that group was 12.4 years (11–14 years). Most common mechanism were lacerations of the wrist (n = 3) and one jump from a height of 3 m with multiple rib fractures.

20 patients (0.16%) were admitted with pathological pain or injury, including 7 fractures without adequate trauma. In all 20 cases, the patients were admitted as inpatients for further diagnosis. The most common locations for pathologic fractures were femur and tibia with 2 patients each.

A total of 174 patients (1.4%) presented to the ED due to pain without trauma. Thereof, 49 patients were treated for torticollis. In the remaining patients, no diagnosis could be made during the examination.

No sufficient documentation regarding injury or trauma mechanism was found in 233 patients.

### Time of presentation

**Fig. 5:**
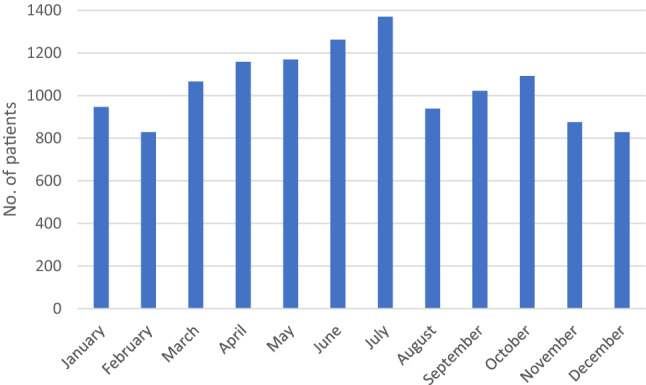
Months of the presentations in the ED

The highest number of presentations in our ED was registered in the months of May, June, and July. In August, there was a significant decrease in the number of presentations (*p* < 0.05) (Fig. 5).

Regarding the days of the week, it was found that the most frequent presentations in the ED were on Fridays, with an average of 7.8 patients. Slightly more patients were seen on weekends (6.9 patients/day) than on weekdays (6.8 patients/day).

**Fig. 6:**
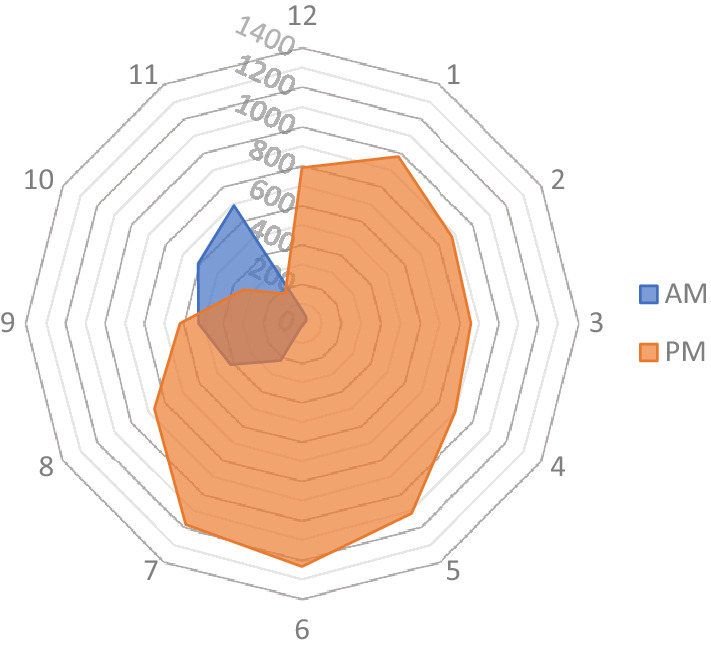
Time of the presentations in the ED

In addition, it showed that an average of 4.0 patients came to the ER in the out-of-office hours between 5 pm and 8 am. Between 8 am and 5 pm, an average of 2.9 patients were treated. Overall, 63% of patients were treated in the out-of-office hours between 5 pm and 8 am (Fig. 6).

## Discussion

In our study, in all age groups male children were more frequently affected by unintentional injuries than females, which is in line with most epidemiological studies [11, 12, 15–17]. This might be associated with a higher exposure to risky sport activities and a different pattern of behavior [12, 16, 18]. The age distribution of patients showed two peaks at ages 1–3 and 10–13 years. Age was hypothesized to have a significant effect on the pattern of physical activity, which in turn affects the injuries associated with physical activity. Ruffing et al. as well as Voth et al. described similar results with peaks in early ages as well as teenagers [16, 17]. In our study, leisure activities, including playground, showed a peak within preschoolers and school-aged children up to 10 years of age. A decrease in incidence was subsequently observed. In older adolescents over 11 years of age, the most common cause of accidents was sport-related activities, which was also observed in other studies [19, 20]. Similar results were found for accidents at home. Al Rumhi et al. and Chini et al. reported that children aged 1–6 and 1–5, respectively, were more likely to visit the emergency department because of accidents at home [21, 22]. Many studies suggest falls as main mechanism for unintentional injuries, especially in young children. This includes falls from low heights or while walking or running [12, 19, 23]. In our study, falls while walking or running were included in leisure activities. Falls from objects, like climbing frames, chairs, and stairs, are a common mechanism in among all ages and often describe the most common cause of accidents in children [12, 19, 20]. With regard to adolescents, many studies describe an increasing incidence of RTA as pedestrians or passengers with high mortality [13, 24]. Especially in developing countries, RTA is described as a major cause of injuries and mortality in older children and adolescents [14, 24, 25]. Interestingly, in our study, no significant increase in injuries associated with RTA at older ages can be seen. In fact, even at the age of 11–14 years, a slight decrease in injuries due to RTA is found. Gong et al. showed similar results with the highest incidence of RTA at the ages between 3 and 6 [26]. The reason for this is probably that many studies include children and adolescents up to 18 or 20 years of age. It is believed that it is only at this age that patients start using motorized vehicles and are more likely to be involved in a traffic accident [7, 10, 24]. Although not the most common mechanism of injury, all deaths in this study are associated with road traffic accidents. Most studies suggest RTA as mechanism with the highest mortality rate among children and young adults with a mortality rates between 0.3% and 8.5% [7, 11–14]. In this study, the rate of severely injured children with an ISS > 16 was low. A comparable study from Germany with 15.300 patients reported an overall rate of children with an ISS > 16 of 0.5% [16]. Furthermore, the mortality rate in the present study was only 0.03% with 4 deaths, which is significantly lower than in comparable studies. Since our hospital, as a level 1 trauma center, is the only one in the nearby area that treats severely injured children, it is difficult to explain the low mortality rate. The hospital is not located in a major city which could lead to lower rate of fatal RTA due to less traffic in general. Additionally, children over the age of 14 are excluded in this study. It is assumed that patients at that age might be less likely involved in accidents in or on motorized vehicles, as already mentioned.

In terms of injuries, all deaths associated with RTA were caused by severe brain injury. This goes in line with most studies saying that the head is the most vulnerable body part, especially in young children [11, 12, 17, 26]. In the present study, the head was the most common body part for lacerations and second most for bruises. It has been shown that especially the disproportionately large head is affected more frequently in young children than in advanced age [16, 17]. Fractures were third most common injuries with peak incidences in school-aged children. Rennie et al. and Randsborg et al. show in a large epidemiological study about fractures in children a bimodal distribution with peaks at the ages of 6–8 and 10–14 with a significant drop in girls over the age of 12 [27, 28]. Similar results were found in our study with a decline in the incidence of girls over 11 years. Furthermore, the prevalence of the various fractures was remarkably similar between the study by Ruffing et al. and the present study. Thus, the distal radius/ulna followed by the metacarpal and fingers were found to be the most common locations of fractures in children [17]. Most common mechanisms for fractures were leisure activities as well as falls. This goes in line with epidemiological studies [19, 27, 29], whereas some studies suggest RTA as most common mechanism resulting in fractures in children [11]. Concerning sports-related activities, Randsborg et al. mentions soccer as being the most dangerous sport with high incidences of distal radial fractures [28]. Not surprisingly, the most common fractures requiring surgical treatment were forearm and humerus fractures. Further injuries were joint dislocations with the nursemaids elbow being the most common one with the highest prevalence at the age between 1 and 3 years. Similar to previous studies, female predominance was found with a ratio of 1.76:1 [30, 31]. Second most common dislocation was the patella dislocation. Studies suggest that between 50 and 70% of patellar dislocations occur while exercising or during other sports-related activity [32, 33]. We found similar results in the present study with the highest prevalence in sports-related activities.

Seasonal as well as time differences could be found in the study. In average, more patients were treated on the weekends than on weekdays. Most patients were admitted to the ED during the late afternoon and early evening hours. In fact, a large percentage of patients were treated in the out-of-office hours. Naqvi et al. reported similar findings with considerably more admissions during the evening with the highest levels of attendance between 5 and 8 pm [11, 19]. Randsborg et al. suggested that the time of admission relates with the season. They found that most admissions during the winter season are around noon, while most admissions in summer are in the late afternoon [28]. In the present study, most admissions were during spring and summer months between May and July. A significant drop in admissions was found in August, which could be explained by the school holidays and many families traveling during that time. Similar results were reported by Ruffing et al. [17].

Suspicion of child abuse was raised in 4 cases and shows an overall low incidence compared to similar studies. Naqvi et al. reported an incidence of 3.8% of suspected child abuse in their study [11]. All patients with suspected child abuse showed typical injuries around the head and uncommon fractures for the age. Typical characteristics described in the literature include patient age less than 1 and head injuries and strangulation marks that may indicate possible child abuse [34, 35].

This study has several limitations. This was a retrospective single-center study. Therefore, the epidemiological data are limited to one area. Some merging of variables had to be done to make the amount of data manageable. This could have resulted in loss of important information. Although the total number of patients is large, some subgroups are small and thus can only be insufficiently included for statistical calculations.

## Conclusion

Most injuries in children are minor in nature and do not require further surgical treatment. The pattern of injury as well as the mechanism of injury varies between the different age groups. Head injuries remain the most dangerous injuries with a high mortality rate in all ages. The leading cause of fatal trauma was traffic accidents. Although in most cases the mechanism is obvious, suspicion of child abuse should be kept in mind. Peak times in the late afternoon and evening and summer months should be taken into consideration of personnel planning.
